# The Generalist Inside the Specialist: Gut Bacterial Communities of Two Insect Species Feeding on Toxic Plants Are Dominated by *Enterococcus* sp.

**DOI:** 10.3389/fmicb.2016.01005

**Published:** 2016-06-28

**Authors:** Cristina Vilanova, Joaquín Baixeras, Amparo Latorre, Manuel Porcar

**Affiliations:** ^1^Cavanilles Institute of Biodiversity and Evolutionary Biology, Universitat de ValènciaValencia, Spain; ^2^Institute for Integrative Systems Biology (I2SysBio), University of Valencia-CSICValencia, Spain; ^3^Unidad Mixta de Investigación en Genómica y Salud, Centro Superior de Investigación en Salud PúblicaValencia, Spain

**Keywords:** **:** lepidoptera, gut communities, metagenomics, *Enterococcus* sp., secondary metabolites

## Abstract

Some specialist insects feed on plants rich in secondary compounds, which pose a major selective pressure on both the phytophagous and the gut microbiota. However, microbial communities of toxic plant feeders are still poorly characterized. Here, we show the bacterial communities of the gut of two specialized Lepidoptera, *Hyles euphorbiae* and *Brithys crini*, which exclusively feed on latex-rich *Euphorbia* sp. and alkaloid-rich *Pancratium maritimum*, respectively. A metagenomic analysis based on high-throughput sequencing of the 16S rRNA gene revealed that the gut microbiota of both insects is dominated by the phylum Firmicutes, and especially by the common gut inhabitant *Enterococcus* sp. *Staphylococcus* sp. are also found in *H. euphorbiae* though to a lesser extent. By scanning electron microscopy, we found a dense ring-shaped bacterial biofilm in the hindgut of *H. euphorbiae*, and identified the most prominent bacterium in the biofilm as *Enterococcus casseliflavus* through molecular techniques. Interestingly, this species has previously been reported to contribute to the immobilization of latex-like molecules in the larvae of *Spodoptera litura*, a highly polyphagous lepidopteran. The *E. casseliflavus* strain was isolated from the gut and its ability to tolerate natural latex was tested under laboratory conditions. This fact, along with the identification of less frequent bacterial species able to degrade alkaloids and/or latex, suggest a putative role of bacterial communities in the tolerance of specialized insects to their toxic diet.

## Introduction

Plants have biochemical and molecular mechanisms to defend themselves from insects attack. Among those, plants produce a vast range of secondary metabolites with anti-herbivore effects, which are produced either constitutively or in response to tissue damage (War et al., 2012). Some plant biochemicals are toxic, repellent, or antinutritive for herbivores. Among these compounds, alkaloids, terpenoids, and complex mixtures of macromolecules such as latex are among the most frequent plant biochemical defense barriers.

Plant alkaloids, are toxic to a wide range of insects (Nuringtyas et al., 2014). However, a few species of insects are unaffected by even high concentrations of alkaloids. The Amaryllidaceae, with more than 300 alkaloids isolated to date (Bastida et al., 2011), are among the most deterrent plants. Alkaloids present in *Pancratium maritimum* have demonstrated both cytotoxic and antimicrobial activity (Hetta and Shafei, 2013). The aposematic larvae of the noctuid moth *Brithys crini* –the “lily borer”– are well known to feed monophagously on the sand lily *Pancratium maritimum*. Sequestration of alkaloids by this species has never been tested but the larvae of the closely related species complex *Xanthopastis timais* – the “Spanish moth”, another Amaryllidaceae specialist – was early included by Rothschild (1973) in her seminal work on insect chemical defense and is a typical example of sequestration of phenanthridine alkaloids (Nishida, 2002). Terpenes are chemical compounds that are present in large amounts in a large variety of plants: in conifers, for example, they are the main components of resin. Plant terpenes are involved in defense against herbivory, even at the belowground level (Vaughan et al., 2013). Terpenes, along with alkaloids, natural gum and many other compounds, are also present in Euphorbiaceae and other plants exuding latex. Unsurprisingly, latex-producing plants are particularly resistant to many insects and other pests (Hagel et al., 2008). The larvae of the sphingid moth *Hyles euphorbiae* – the “spurge hawk moth” – feed on a broad variety of *Euphorbia* plants from which they sequester the cytotoxic ingenane diterpene esters (Marsh and Rothschild, 1984).

Beyond biochemicals, plant defenses against herbivory also involve mutualistic microorganisms. Endophytic fungi, plant symbionts living asymptomatically within the host tissues, produce alkaloid-based herbivore deterrents that contribute to the defense of the plant (Koh and Hik, 2007). Reciprocally, bacteria associated with insects can play a role in disturbing plant defensive barriers (Hansen and Moran, 2014; Hammer and Bowers, 2015). For example, Colorado potato beetle (*Leptinotarsa decemlineata*) larvae have been reported to bear bacteria in their oral secretions that suppress antiherbivore defenses in tomato (*Solanum lycopersicum*), the plant the beetle feeds on (Chung et al., 2013). In summary, plant–insect interactions are complex ecological processes mediated by secondary metabolites, alkaloids and terpenes among them, but also by microorganisms, which play key roles as both defense and attack allies for the plants and phytophagous insects, respectively. Surprisingly enough, though, there are few reports on that topic. The few studies available on the microbial communities associated to the gut of insects, and particularly to Lepidoptera, are focused on species considered as agricultural or forest pests worldwide. This is the case of the gypsy moth *Lymantria dispar* (Broderick et al., 2004; Mason and Raffa, 2014), the diamondback moth *Plutella xylostella* (Lin et al., 2015), or the cotton bollworm *Helicoverpa armigera* (Xiang et al., 2006). Also, some focus has been put on how diet changes influence the gut microbiota of polyphagous insects such as *Bombyx mori* (Liang et al., 2014), *Spodoptera littoralis* (Tang et al., 2012), or *Ostrinia nubilalis* (Belda et al., 2011).

**FIGURE 1:**
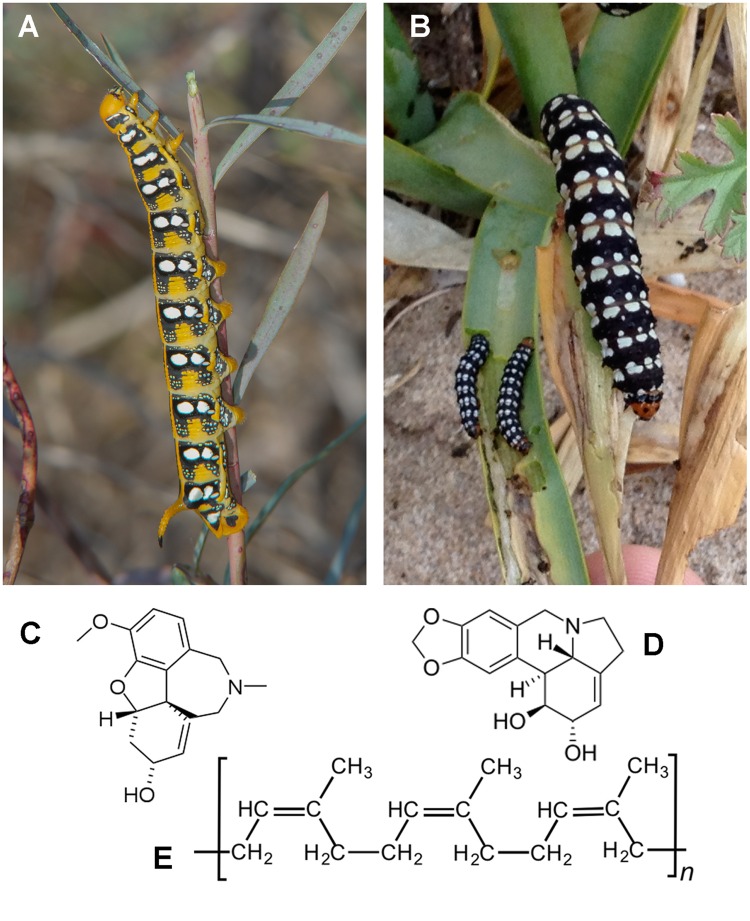
**Larvae and representative secondary compounds found in the diet.** **(A)** *Hyles euphorbiae*; **(B)** *Brithys crini*; **(C)** galanthamine (*Pancratium maritimum*); **(D)** narciclasine (*P. maritimum*); **(E)** latex (*Euphorbia* sp.).

Latex- and alkaloid-rich plants constitute a particularly strong selection pressure not only for phytophagous insects (Kirk et al., 2012; Ramos et al., 2015), but also for their gut microbiota, which is subjected to a constant flow of toxic compounds. The gut of insects feeding on toxic plants is thus a unique and extreme habitat. We present here a complete characterization of the bacterial gut symbionts of two monophagous Lepidoptera feeding on plants rich in, among other toxic compounds, a cocktail of alkaloids or terpenes (**Figure 1**). This is the first report of the microbial larval gut communities associated to such toxic diets.

## Materials and Methods

### Sampling

Larvae of *B. crini* and *H. euphorbiae* (**Figure 1**) were obtained in the field (coastal dunes from Pinedo and El Saler, Valencia, Spain) by direct inspection of the food plants *P. maritimum* and *Euphorbia* sp., respectively, in the adequate moment of the year: spring for most larvae of *B. crini* and autumn for *H. euphorbiae*.

### Gut Dissection and DNA Extraction

Last instar larvae from *B. crini* and *H. euphorbiae* were kept in starvation for one day to promote the elimination of plant material from the gut. Larvae were immobilized by placing them on ice and both the midgut and hindgut were dissected under sterile conditions. Guts from three different individuals of each species were independently disaggregated and manually homogenized in PBS buffer (NaCl 8 g/L, KCl 0.2 g/L, Na_2_HPO_4_ 1.44 g/L, and KH_2_PO_4_ 0.24 g/L, pH adjusted to 7.4) with an Eppendorf-adapted pestle. Total DNA was obtained from the homogenate with a standard purification protocol consisting of alkaline lysis followed by precipitation with potassium acetate and isopropanol (Latorre et al., 1986). An initial incubation step with 2 μg/mL lysozyme at 37°C for 30 min was performed to ensure the lysis of Gram-positive bacteria. The quality of the DNA was finally checked on a 0.8% (w/v) agarose gel and quantified with Nanodrop-1000 Spectophotometer (Thermo Scientific, Wilmington, DE, USA).

### PCR Amplification and 16S rRNA Profiling

A 700 bp fragment of the V1–V3 hypervariable region of the 16S rRNA genes was polymerase chain reaction (PCR)-amplified from all the samples with universal primers 28F (5′-GAG TTT GAT CNT GGC TCA G-3′) and 519R (5′-GTN TTA CNG CGG CKG CTG-3′). A short (9–11 nucleotides) barcode sequence followed by a four-nucleotide spacer (CGAT) was included at the 5′ end of the oligonucleotides used as forward primers to enable assignment of sequences to samples after high-throughput sequencing. All the amplifications were performed under the following thermal cycling conditions: initial denaturing at 95°C for 5 min, followed by 35 cycles of denaturing at 95°C for 30 s, annealing at 55°C for 30 s, and extension at 72°C for 1 min, finalized by a 10-min elongation at 72°C. Amplicons were checked on a 0.8% (w/v) agarose gel and purified by precipitation with 3 M potassium acetate (pH 5) and isopropanol. Pure amplicons were quantified with the Qubit^®^ 2.0 Fluorometer (Invitrogen, Carlsbad, CA, USA) and an equimolar pool of amplicons was prepared from all the samples.

Amplicons of the 16S rRNA gene for all the samples were pyrosequenced with a Roche GS FLX sequencer and Titanium chemistry in the Center for Public Health Research (FISABIO-Salud Pública, Valencia, Spain). All the sequences obtained were split into groups (a maximum of two mismatches were allowed for primer search, whereas no mismatches were allowed for barcode search), trimmed with a minimum quality score of 20, and filtered to remove short reads (<150 nt). Then, sequences were clustered and taxonomically assigned with the open-reference operational taxonomic unit (OTU) picking pipeline implemented in the QIIME software (Caporaso et al., 2010). Clustering was performed at a similarity threshold of 95% (genus-level OTUs) and the 16S rRNA Greengenes database (version 13_8) was used as reference. Finally, the resulting OTU table was processed and analyzed with software MEGAN (Huson et al., 2011). A summary of statistics is available as Supplementary Table S1. Sequences were deposited in the MG-RAST public repository under accession numbers 4639004.3–46390015.3.

### Scanning Electron Microscopy

Small fragments (2–5 mm) of *B. crini* and *H. euphorbiae* hindguts were dissected. Fragments were fixed by immersion into paraformaldehyde 2% – glutaraldehyde 2.5% for more than 2 h, washed with water and refixed by osmium tetroxide for 20 min, washed and dehydrated in absolute ethanol. These pieces were placed inside microporous capsules (30 μm pore size, available from Ted Pella Inc. product number 4619) immersed in absolute ethanol, following critical point drying in an Autosamdri 814 (Tousimis). Dry samples were then arranged on SEM stubs with silver conducting paint TAAB S269. Pieces were manipulated under a stereomicroscope Leica MZ9.5 with Dumont forceps number 5. Stubs were examined under a scanning electron microscope Hitachi S-4100. Images were edited with Photoshop CS3 (Adobe).

### Metagenomic Sequencing of the Bacterial Biofilm

The biofilm of a particular *H. euphorbiae* specimen was dissected. Total DNA was isolated with the same protocol described above and then subjected to shotgun metagenomic sequencing in the Center for Public Health Research (FISABIO-Salud Pública, Valencia, Spain). A Nextera Illumina library was built from 100 ng of total DNA following the protocol indications by Illumina. The library was sequenced in a MiSeq sequencer (Illumina) in a combination of 500 cycles, in order to obtain 250 bp paired-end sequences. The MG-RAST platform (Meyer et al., 2008) was used to filter out sequences matching the insect’s genome and to taxonomically classify the 16S rRNA sequences belonging to bacteria. To do that, sequence similarity searches were performed against the non-redundant m5RNA database (**Supplementary Figure S1**). Sequences were deposited in the MG-RAST public repository under accession numbers 4633589.3.

### Culture Media and Growth Conditions

The *E. casseliflavus* strain isolated from *H. euphorbiae* hindgut was maintained on LB medium (10 g/L NaCl, 10 g/L bacteriological peptone, 5 g/L yeast extract; and 15 g/L agar for solid medium) at room temperature. The ability of the strain to tolerate or degrade latex was tested in both LB and artificial minimal synthetic medium (2 g/L NaNO_3_, 1 g/L K_2_HPO_4_, 0.5 g/L MgSO_4_, 0.5 g/L KCl, 0.2 g/L bacteriological peptone; and 15 g/L agar for solid medium) supplemented with 10–20% (v/v) *Euphorbia* sp. plant extract or 1–3% (v/v) natural liquid latex (Chemionics Corp., Tallmadge, OH, USA) as the sole carbon sources, respectively, at room temperature. Plant extracts enriched in latex were obtained by grinding 300 g *Euphorbia* sp. with a domestic blender. The mixture was infused overnight with 150 ml of pure ethanol and then filtered through Whatmann paper. The resulting raw plant extracts were added to the sterilized media at 65–70°C. The ability of the resulting media to inhibit bacterial growth was tested in *Escherichia coli* XL1-Blue strain, which proved unable to grow in the presence of either the plant extract or the natural latex.

### Identification of *E. casseliflavus* Strain He

A colony PCR was performed to identify the taxonomy of the *Enterococcus* strain isolated from *H. euphorbiae* gut. A fragment of the 16S rDNA gene was amplified with universal primers 28F (5′-GAG TTT GAT CNT GGC TCA G-3′) and 519R (5′-GTN TTA CNG CGG CKG CTG-3′). The PCR program was as follows: an initial denaturing step at 95°C for 300 s, followed by 35 cycles of denaturing, annealing and extension (95°C, 30 s; 48°C, 30 s; and 72°C, 60 s) and a final extension step at 72°C for 480 s. PCR amplicons were purified by the High Pure PCR Product Purification Kit (Roche Diagnostics GmbH, Mannheim, Germany) and sequencing was carried out with the ABI PRISM BigDye Terminator v3.1 system (Applied Biosystems) on an ABI 3730 automated sequencer. PCR products were sequenced in both senses with the 28F and 519R primers. Sequences were verified and both strands assembled using the STADEN package. Sequence taxonomy was attributed with BLASTN searches against the RefSeq database of the NCBI. The closest match corresponded to *E. casseliflavus* strain RTCLI14 (sequence similarity = 99%; *e*-value = 5*e* - 115).

## Results

**FIGURE 2:**
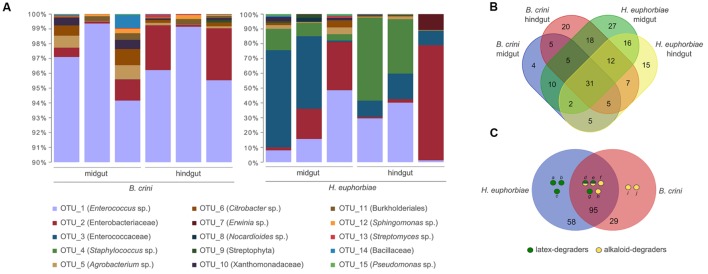
**Bacterial composition of *B. crini* and *H. euphorbiae* guts as deduced by massive 16S rRNA sequencing.** **(A)** Relative abundance of bacterial operational taxonomic units (OTUs) in the midgut and hindgut sections of three different individuals of each species. Note that the scale of *Y*-axis starts at 90% for *B. crini* samples. **(B)** Venn diagram showing overlapping and exclusive OTUs occurring in *B. crini* and *H. euphorbiae* guts. **(C)** Occurrence of potential latex-degrading and alkaloid-degrading bacteria in the guts of *B. crini* and *H. euphorbiae* in correlation with their diet (a: *Nocardioides* sp.; b: *Gordonia* sp.; c: *Curtobacterium* sp.: d: *Pseudomonas* sp.; e: *Bacillus* sp.; f: *Sphingomonas* sp.; g: *Streptomyces* sp.; h: *Propionibacterium* sp.; i: *Klebsiella* sp.; and j: *Corynebacterium* sp.).

The bacterial composition of gut extracts from triplicates of larvae of *B. crini* and *H. euphorbiae* was investigated by high throughput sequencing of the 16S rRNA amplicons. A total of 182 species-level OTUs representing 87 different genera were detected in total. As **Figure 2A** shows, Firmicutes (OTUs 1, 3, and 4) were, by far, the most abundant bacterial taxa in all cases. Nevertheless, the overall taxonomic profiles from the two species exhibited a clear difference: whereas *B. crini* was characterized by the overwhelming presence of *Enterococcus* sp., (OTU 1, accounting for 94–99% of reads), *H. euphorbiae* harbored a more heterogeneous community. *Enterococcus* sp. was found at high frequencies (10–50%), and another species of the Enterococcaceae family (OTU 3) was also detected at similar frequencies (10–60%). A species belonging to the Enterobacteriaceae family (OTU 2) and *Staphylococcus* sp. (OTU 4) were very common in *H. euphorbiae* (8–70% of sequences depending on the specimen) but were rare in *B. crini*. In that species, the bacterial composition of both midgut and hindgut sections of the insect gut were similar, although some differences were detected (see below). In the case of *H. euphorbiae*, midgut samples exhibited higher amounts of Enterococcaceae (OTU 3) in comparison to hindgut samples, which were richer in *Staphylococcus* sp. and enterobacteria (**Figure 2A**).

The remaining bacterial taxa were detected at very low levels in both insects and in both the medium and final sections of the gut. Of these, 58 were exclusive of *H. euphorbiae* (of which 27 were exclusive of the midgut and 15 were exclusive of the hindgut); 29 were exclusive of *B. crini* (20 of which were exclusive of the hindgut); and 95 were found in both species in at least one sample. **Figure 2B** shows a Venn diagram with overlapping and exclusive genera occurring in *B. crini* and *H. euphorbiae* samples.

A systematic bibliographic search was made with bacterial taxa (those which could be identified to the level of genus or species) in order to identify alkaloid- or latex-degrading abilities. As **Figure 2C** shows, we identified minoritary taxa (accounting for near 1% of the total number of *reads*) reported to have latex and/or alkaloid degradation abilities in both insect species. Three latex degraders (*Nocardioides* sp., *Gordonia* sp., and *Curtobacterium* sp.) were exclusively present in *H. euphorbiae*, whereas two alkaloid degraders (*Klebsiella* sp. and *Corynebacterium* sp.) were detected only in *B. crini*.

**FIGURE 3:**
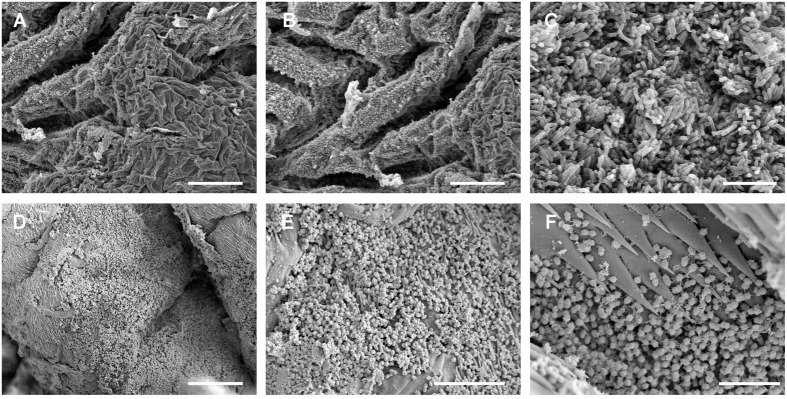
**Scanning electron microscope images of the luminal surface of the hindgut of *B. crini***(A–C)** and *H. euphorbiae***(D–F)**.** A biofilm of bacilli was observed in some *B. crini* specimens, whereas a biofilm of cocci was detected in all the *H. euphorbiae* specimens analyzed. Notice the presence of acanthae around the biofilm area in *H. euphorbiae*. Scale bars: **(A,B)** = 50 μm; **(C)** = 7 μm; **(D)** = 25 μm; **(E)** = 10 μm; **(F)** = 5 μm.

Electron micrographs of the gut surface of five individuals of both species showed a virtual absence of bacteria in the midgut. However, some *B. crini* individuals and all the *H. euphorbiae* specimens analyzed showed a detectable ring-like layer of bacteria at the level of the pyloric valve (**Figure 3**) of the hindgut. Bacteria concentrated in both species on more sclerotized areas and were particularly common around acanthae. An *H. euphorbiae* hindgut sample with a particularly dense bacterial layer was subjected to total DNA isolation and metagenomic sequencing, which allowed the identification of the most frequent bacterium of the biofilm ring as *Enterococcus casseliflavus* (**Supplementary Figure S1**). This *E. casseliflavus* strain (hereafter called *E. casseliflavus* He) was isolated in pure culture. In order to check that this isolate corresponded to the dominant bacterium of the biofilm, the 16S rRNA gene was sequenced and confirmed to be more than 99% identical to the most abundant 16S rRNA gene detected through the metagenomic sequencing of the ring. Also, the sequence was found to be more than 99% identical to the representative 16S rRNA sequence of OTU 3 (the Enterococcaceae species detected at moderate abundance in *H. euphorbiae*). *E. casseliflavus* He exhibited strong growth on media supplemented either with natural latex or *Euphorbia* sp. plant extract, but failed to use natural latex as the sole carbon source (data not shown), suggesting that this strain is able to tolerate – rather than grow on – latex molecules.

## Discussion

The gut microbial communities of two lepidopteran species feeding on toxic plants rich in latex and alkaloids are examined for the first time. Although *B. crini* and *H. euphorbiae* belong to different Lepidopteran families (Noctuidae and Sphingidae, respectively), they proved to harbor similar bacterial communities, surprisingly dominated by the bacterium *Enterococcus* sp., and to a lesser extent in the case of *H. euphorbiae*, by other Enterococcaceae species, an enterobacterium, and *Staphylococcus* sp. The taxonomic profile of midgut and hindgut samples did not significantly differ in *B. crini*, but showed some differences in *H. euphorbiae*. This, as well as the scarce presence of bacteria detected through SEM in midgut samples of both species, might be a consequence of the strong alkaline pH of the midgut of Lepidoptera (Dow, 1992), even though the particular pH conditions of the insects analyzed in this study have not been determined to date. Additionally, a range of genera containing species which are known to degrade latex and/or alkaloids were detected in *H. euphorbiae* and *B. crini*, respectively, which is in correspondence with their diets. Among these, genus *Pseudomonas* is known to harbor several species able to degrade alkaloids (*P. putida* and other unidentified isolates) and natural latex or rubber (*P. aeruginosa* and *P. citronellolis*); and genus *Streptomyces* is considered especially rich in latex- and rubber-degrading species (*S. coelicolor*, *S. griseus*, *S. lividans*, etc.; Jendrossek et al., 1997; Bode et al., 2001; Rathbone and Bruce, 2002 and references therein).

Bacteria belonging to the genera *Enterococcus* and *Staphylococcus* are prevalent in Lepidoptera of the families Sphingidae and Noctuidae (Visôtto et al., 2009), and have been traditionally considered generalist bacteria, since they are widely present in insects (Martin and Mundt, 1972; Tholen et al., 2006; Geiger et al., 2009; Tang et al., 2012). For instance, *E. casseliflavus* has recently been isolated from the lepidopteran *Spodoptera litura*, a highly polyphagous major pest on many crops (Thakur et al., 2015), and also from *Manduca sexta* (Lepidoptera, Sphingidae), a specialist species that feeds on toxic Solanaceae, rich in phenolic derivatives of caffeic acid (Brinkmann et al., 2008).

The formation of biofilms dominated by a single bacterial species, such as the one we report in this work, in insect’s gut has been traditionally related to entomopathogenic bacteria (Vodovar et al., 2006; Vallet-Gely et al., 2008), although some studies have demonstrated that biofilm formation is essential for the establishment of symbiotic relationships between bacteria and the host insect (Maltz et al., 2012; Vásquez et al., 2012). In particular, *E. casseliflavus* has been found associated with larvae of *Spodoptera litura* (Lepidoptera, Noctuidae) feeding on lima beans, which are especially rich in toxic terpenes such as carotenes. In this case, *E. casseliflavus* forms a monospecific biofilm in which toxic alpha- and beta-carotenoids are crystallized, and larvae failing to develop the biofilm exhibit increased mortality (Shao et al., 2011). Given the similarity between carotenes and latex in terms of chemical structure, and the ability of *E. casseliflavus* He to tolerate latex, it is tempting to hypothesize that this strain might be involved in latex immobilization in *H. euphorbiae* hindgut.

This work describes and sheds light in a putatively new case of close relationship between an – apparently – generalist bacterium and a specialist insect. The microbiota described in this work, and especially the *E. casseliflavus* He strain isolated from *H. euphorbiae* hindgut, may be of interest not only for understanding the ecology of such specialist insects, but also for the biotechnological industry, where microorganisms and/or enzymes able to transform alkaloids or latex-like molecules may have biotechnological applications such as bioremediation.

## Author Contributions

AL, MP, JB, and CV designed the study. CV and JB performed the sampling. JB conducted the electron microscopy experiments. CV and MP analyzed the data. AL, MP, JB, and CV wrote and revised the manuscript.

## Conflict of Interest Statement

The authors declare that the research was conducted in the absence of any commercial or financial relationships that could be construed as a potential conflict of interest.

## Supplementary Material

**FIGURE S1 | Taxonomic classification of the metagenomic sequences from the biofilm ring.** The species-level assignations of sequences belonging to the 25 bacterial domain are depicted on the right.
