# Supplementary figures and images for: The Generalist Inside the Specialist: Gut Bacterial Communities of Two Insect Species Feeding on Toxic Plants Are Dominated by Enterococcus sp
Source: Front Microbiol. 2016 Jun 28;7:1005. doi: 10.3389/fmicb.2016.01005 (PMC4923067; doi:10.3389/fmicb.2016.01005)

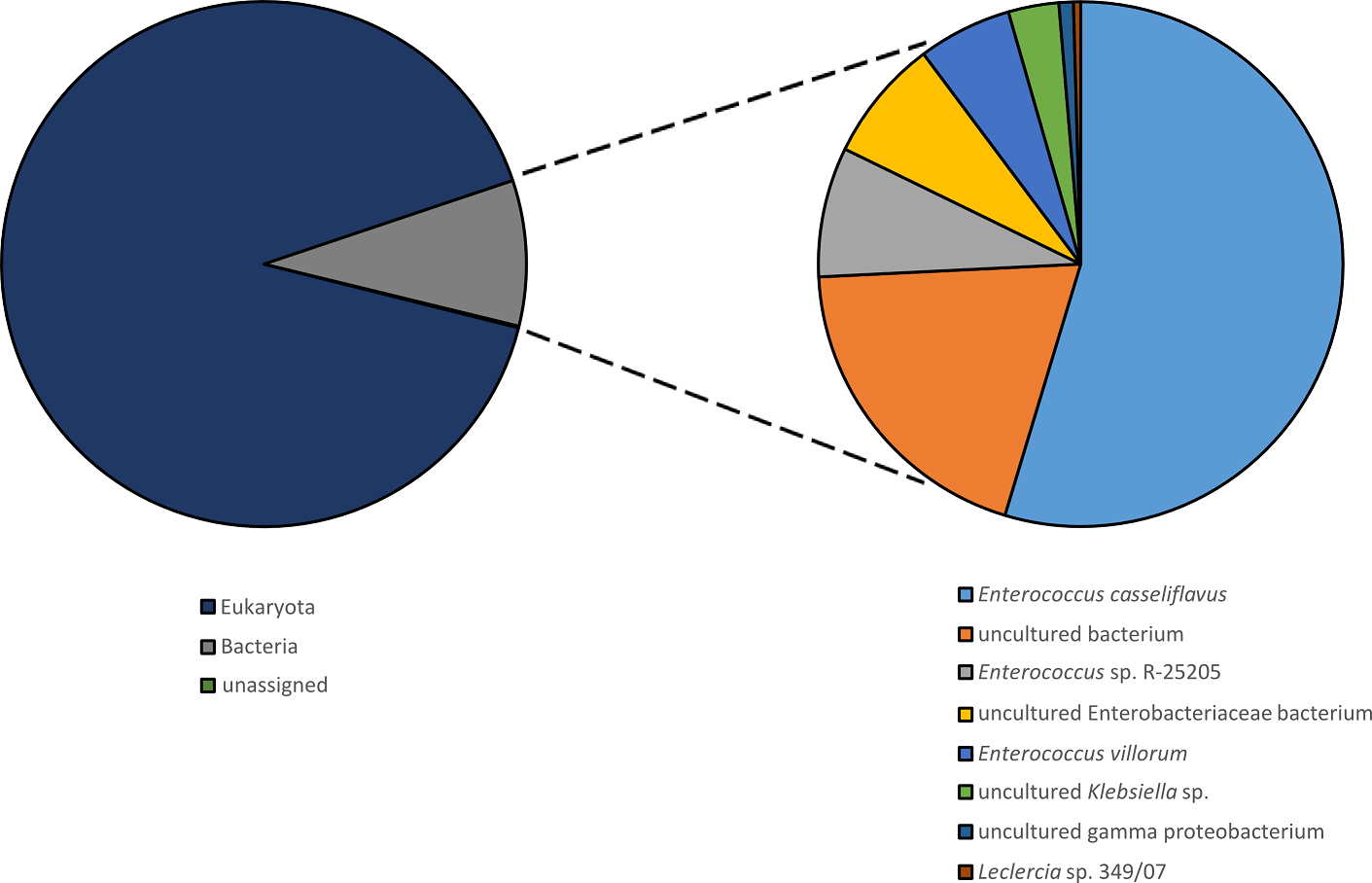

Supplement: Supplementary file 1 [file Image_1.TIF]
