# Supplementary material for: The Generalist Inside the Specialist: Gut Bacterial Communities of Two Insect Species Feeding on Toxic Plants Are Dominated by Enterococcus sp
Source: Front Microbiol. 2016 Jun 28;7:1005. doi: 10.3389/fmicb.2016.01005 (PMC4923067; doi:10.3389/fmicb.2016.01005)
Supplement: Supplementary file 2 [file Table_1.DOCX]

| Sample* | Raw reads | Reads after trimming | Observed Species | 1/Simpson index |
| --- | --- | --- | --- | --- |
| Bc_MG_I | 6186 | 6167 | 73 | 1,14 |
| Bc_MG_II | 11831 | 11821 | 50 | 1,05 |
| Bc_MG_III | 6861 | 6825 | 108 | 1,26 |
| Bc_HG_I | 18712 | 18659 | 78 | 1,12 |
| Bc_HG_II | 21693 | 21591 | 79 | 1,05 |
| Bc_HG_III | 31313 | 31209 | 114 | 1,14 |
| He_MG_I | 220 | 213 | 16 | 7,56 |
| He_MG_II | 15921 | 15559 | 259 | 3,57 |
| He_MG_III | 3971 | 3848 | 209 | 3,74 |
| He_HG_I | 2073 | 2056 | 56 | 2,90 |
| He_HG_II | 7660 | 7542 | 222 | 3,51 |
| He_HG_III | 4118 | 4090 | 62 | 1,76 |

**Supplementary Table 1.** Number of reads and diversity estimates for the samples analyzed.

* Bc: *B. crini*; He: *H. euphorbiae*; MG: midgut; HG: hindgut
